# Observance thérapeutique de l’hypertension artérielle et ses facteurs dans le service de cardiologie du CHU Tokoin de Lomé

**DOI:** 10.11604/pamj.2013.14.48.1973

**Published:** 2013-02-04

**Authors:** Machihude Pio, Soodougoua Baragou, Yaovi Afassinou, Souleymane Pessinaba, Borgatia Atta, Koffi Ehlan, Edem Goeh-Akue, Folly Zoméni, Jean-Marie Damorou

**Affiliations:** 1 Faculté Mixte de Médecine et de Pharmacie, Université de Lomé, Togo

**Keywords:** Hypertension artérielle, observance thérapeutique, Togo, hypertension, adherence, Togo

## Abstract

**Introduction:**

Evaluer l’observance thérapeutique de l’HTA et de déterminer les facteurs de mauvaise observance chez les hypertendus au CHU Tokoin de Lomé.

**Méthodes:**

Nous avons effectué une étude transversale descriptive sur une durée de 6 mois en consultation externe dans le service de Cardiologie du CHU-Tokoin de Lomé. L’étude a concerné 363 patients hypertendus. L’évaluation de l’observance a été effectuée avec le test d’évaluation de l’observance de Girerd.

**Résultats:**

Le revenu mensuel était inférieur à 50.000 FCFA chez 49,86% patients; 85,05% des patients n’avaient aucune assurance maladie. Le coût mensuel du traitement par patient était de 10.560 FCFA soit 37,74% du SMIG au Togo. Quatre-vingt et trois virgule soixante-quinze pour cent (83,75%) de nos patients avaient eu des difficultés à observer correctement leur traitement antihypertenseur. Parmi eux 52,34% étaient mauvais observants et 31,41% avaient de minimes problèmes d’observance. Seuls 16,25% étaient de bons observants. Les facteurs de mauvaises observances étaient: le revenu mensuel faible, l’absence d’assurance maladie, le nombre de comprimés et de prises de comprimés supérieurs à 3, et la tradithérapie.

**Conclusion:**

L’observance thérapeutique a été mauvaise dans notre série. Les facteurs de cette mauvaise observance sont multiples et variés mais dominés pour la plupart par la pauvreté et l’absence d’assurance maladie.

## Introduction

L’observance thérapeutique de l’hypertension artérielle (HTA) se définit comme le respect de la prescription médicamenteuse et non médicamenteuse par le patient souffrant de l’HTA [1]. Les pathologies à évolution chronique imposant un traitement au long cours avec de nombreux changements de comportement de vie qui sont associées à une mauvaise observance du traitement entrainant des effets délétères pour le patient comme pour la collectivité. Faciliter l’observance est donc un enjeu important. Seulement moins du tiers des patients hypertendus traités sont normalisés dans la plupart des pays. [2] De rares données sur l’observance thérapeutique existent au Togo. D’où l’intérêt de notre étude qui a pour objectif de déterminer les facteurs de mauvaises observances thérapeutiques chez les hypertendus au Togo admis au CHU-Tokoin.

## Méthodes

Nous avons effectué une étude transversale dans le service de cardiologie du Centre Hospitalier Universitaire Tokoin de Lomé (CHU-T) de janvier 2010 à décembre 2011. Elle a porté sur les patients hypertendus suivis depuis plus de 3 mois au cours de la période d’étude et qui ont répondu favorablement au questionnaire du test d’observance (TEO) de Girerd[3]. Les patients dont les dossiers étaient incomplets ne comportant pas d’adresse complète et les patients qui n’ont pas pu répondre au questionnaire n’ont pas été inclus dans l’étude. L’étude s’est déroulée en 2 phases: la 1^ème^ phase est rétrospective. Elle a consisté à collecter les patients suivis pour HTA de plus de 3 mois en consultation externe. Elle a duré 8 jours. La 2^ème^ phase a duré 6 mois. Au cours de cette phase les patients sont invités par téléphone et sont soumis à un questionnaire de TEO mis au point par Girerdet al. Les paramètres étudiés étaient d’ordre épidémiologique, thérapeutique. Au cours de notre période d’étude, 684 patients hypertendus, ont été suivis et seulement 363 (204 femmes et 159 hommes) ont répondu à notre étude. L’âge moyen de notre population était de 50,44 ± 12,33. Les données ont été traitées sur ordinateur à l’aide du logiciel EPI INFO version 6.04 fr.

## Résultats

### Le revenu mensuel

La majorité de nos patients avait un revenu mensuel bas (Tableau 1). La couverture médicale en assurance est faible. Plus de 80% de nos patients hypertendus (87,05%) n’avaient pas d’assurance maladie et seulement 12,95 étaient assurés.

**Tableau 1:** Répartition des patients de l’étude et des patients mauvais observants en fonction de leur revenu mensuel

	Patients de l’étude		Patients mauvais observants	
	Effectif	Pourcentage	Effectif	Pourcentage
<50.000	181	49,86	111	61,33
50.000-100.000	78	21,49	37	47,44
100.000-150.000	56	15,43	30	53,57
>150.000	48	13,22	12	25,00
**Total**	363	100,00	190	52,34

### Modalités thérapeutiques

Au plan thérapeutique, la bithérapie (fixe et séparée) a été plus prescrite chez 52,62%, une monothérapie chez 41,04%, une trithérapie séparée chez 6,34%. La plupart de nos patients prenait plus de deux comprimés par jour, dont 171(47,10%) qui prenaient trois comprimés et plus (Tableau 2). Deux cent vingt et un patients (60,88%) avaient au moins deux prises médicamenteuses par jour dont 196 (53,99%) ayant deux prises journalières et 25 (6,89%) trois prises journalières.

**Tableau 2:** Répartition des patients en fonction du nombre de comprimés pris par jour

	Effectif	Pourcentage
**1**	107	29,48
**2**	85	23,42
**3**	125	34,43
**>3**	46	12,67
**Total**	363	100,00

### Le niveau d’observance

Trois cent quatre (83,75%) avaient des difficultés à observer correctement leur traitement dont 114 (31,41%) ayant de minimes problèmes d’observance et 190 (52,34%) de mauvais observants. Cinquante et neuf (16,25%) étaient de bons observants. Deux cent quatre-vingt et seize patients (80,99%) étaient informés sur leur maladie. Soixante et neuf virgule soixante et sept pour cent des patients connaissaient les complications de leur maladie, 84,02% connaissaient leur régime alimentaire. Soixante et dix-neuf virgule trente et quatre pour cent avaient affirmé être suffisamment informés de leur maladie et appréciaient la qualité de leurs médecins. Soixante et seize virgule quatre-vingt et six pour cent avaient une bonne relation et le soutien de leur entourage.

### Les facteurs de mauvaise observance

Les facteurs de mauvaise observance ont été divers: l’âge, la profession, le revenu mensuel, le traitement et son coût. Les mauvais observants étaient de tous les âges. Les meilleurs observants ont été aux âges extrêmes (moins de 30 ans et plus de 80 ans) avec respectivement 54,54% et 60% de bonne observance. Entre 30 et 80 ans, plus l’âge était avancé, plus l’observance était mauvaise. L’observance thérapeutique était mauvaise presque au même titre chez les femmes que les hommes, avec respectivement 53,92% et 50,31%. Dans notre série d’étude, les femmes au foyer étaient les plus mauvais observants (71,93%) suivis des patients du secteur informel (54,84%). La qualité de l’observance thérapeutique était presque fonction du revenu mensuel (Tableau 1). L’observance était beaucoup plus mauvaise chez les patients en bithérapie et trithérapie avec respectivement 56,02% et 69,57% contre 44,97% des patients en monothérapie. Quatre-vingt et quatorze virgules douze pour cent et 67,39% des patients qui avaient respectivement trois comprimés et plus par jour étaient de mauvais observants. Soixante et seize pour cent et 58,16% de ceux qui avaient respectivement trois et deux prises par jour étaient aussi de mauvais observants. Le coût total du traitement de l’hypertension artérielle était estimé dans notre population à 3.836.390 FCFA par mois soit une moyenne de 10.568,57 FCFA par mois et par patient. De ce fait, 75,48% de nos patients ont jugé leur traitement trop cher, 58,13% ont dû arrêter leur traitement et 24,52% de nos patients ont fait recours à la tradithérapie en remplacement ou en complément de leur traitement.

## Discussion

Notre étude est une étude transversale avec quelques biais engendrés en rapport avec le non-respect des rendez-vous. Toutefois, notre objectif qui était d’évaluer l’observance thérapeutique et de dégager les facteurs de mauvaise observance a été atteint.

Une monothérapie antihypertensive a été prescrite chez 41,04%, une bithérapie chez 52,62% et une trithérapie chez 6,34%. Konin et al [4] en Côte d’Ivoire avaient trouvé 45% des patients chez qui une monothérapie a été prescrite, une bithérapie chez 28,5% et une trithérapie ou plus chez 26,5%.

«Il n’existe aucun moyen parfait pour mesurer l’observance thérapeutique. L’anamnèse n’est pas fiable. Le jugement clinique est peu fiable: les médecins sont trop optimistes et se trompent près de deux sur trois lorsqu’ils veulent détecter les patients non observants» [5]. Le recours au monitoring thérapeutique peut être utilisé pour objectiver l’observance. Le dosage de la concentration sérique ou urinaire du médicament permet alors d’attester la prise régulière du traitement. [6] Pour simplifier l’évaluation de l’observance thérapeutique, Girerdet al [3] ont mis au point un questionnaire standardisé simple qui est un véritable test d’évaluation de l’observance (TEO). Selon ce TEO, nous avions observé une proportion de 16,25% de patients qui avaient une bonne observance thérapeutique, 31,41% de patients qui avaient de minimes problèmes d’observance et 52,34% étaient de mauvais observants. Ces chiffres montrent les difficultés à l’observance thérapeutique de l’HTA dans notre étude. GOLAY et al [7] en France en 2004 avaient trouvé du 50% des patients, toutes maladies confondues ne prenant pas correctement leur traitement. Scheenet al [6] en 2010 avaient estimé le nombre de patients non observants à 30 et 60%. Girerdet al [3] en France avaient trouvé que 66% des patients étaient de bons observants et 10% étaient de mauvais observants. Adoubi et al. [2] et Konin et al. [4] en Côte d’Ivoire avaient trouvé respectivement 26,8% et 12,5% des patients qui étaient de bons observants; 19, 6% et 55% des patients qui étaient de mauvais observants. L’observance thérapeutique semble être bonne dans les pays en développement. En Afrique noire, ces taux élevés de mauvaise observance peuvent s’expliquer par les difficultés économiques et l’absence de couverture médicale.

Les données de la littérature concernant l’influence des facteurs socio-démographiques sur l’observance thérapeutique sont contradictoires [4]. Certains auteurs ont trouvé une association significative entre les facteurs socio-démographiques et l’observance thérapeutique de l’HTA [4]. Nos résultats n’ont pas montré une influence claire de tous les facteurs socio-démographiques sur le niveau d’observance thérapeutique de l’HTA. L’observance thérapeutique était mauvaise dans toutes les tranches d’âge de nos patients. Cependant le taux de mauvaise observance était plus élevé dans la tranche d’âge de 50-60 ans (63, Nous estimons que ce résultat est lié aux exigences de la vie active de ces patients car les patients trop actifs ont des difficultés à se soumettre aux exigences des médecins 4’ > 81%). Nous estimons que ce résultat est lié aux exigences de la vie active de ces patients car les patients trop actifs ont des difficultés à se soumettre aux exigences des médecins [4]. L’observance thérapeutique était mauvaise presque au même titre chez les femmes que les hommes. Nos résultats diffèrent de ceux trouvés par Konin et al [4] où les hommes étaient un peu mieux observants que les femmes. La raison en était que les femmes sont économiquement dépendantes du salaire déjà précaire de leur mari [4]. Dans notre étude les hommes comme les femmes sont confrontés aux mêmes difficultés économiques qui font d’eux de mauvais observants au même titre. Soixante et un virgule trente et un pour cent des patients qui avaient un revenu inférieur à 50.000 FCFA, 53,57% de ceux qui avaient un revenu entre 100.000 FCFA 150.000 FCFA et 47,44% de ceux qui gagnaient entre 50.000 FCFA 100.000 FCFA étaient des mauvais observants. Konin et al. [4] avaient trouvé une mauvaise observance chez 64% des patients qui avaient un revenu mensuel inférieur à 100.000 FCFA et 50% de ceux qui avaient plus de 300.000 FCFA. Il ressort de ce résultat que pour avoir un taux élevé de bonne observance, il faut accroître le pouvoir d’achat des populations. Le coût mensuel global des médicaments du traitement de l’HTA dans notre échantillon était estimé en moyenne à 10539,390 FCA par patient et par mois. Ceci montre le coût encore élevé du traitement de l’HTA eu égard au revenu mensuel bas de la majorité de nos patients. Le nombre de comprimés et de prises journalières avait été un facteur de mauvaise observance. Selon GIRERD, les associations des facteurs de risque cardio-vasculaires obligent les patients à respecter jusqu’à dix lignes d’ordonnance, réduisant ainsi l’observance thérapeutique. Dans cette étude, 94,12% et 67,39% des patients qui avaient respectivement trois comprimés et plus par jour étaient de mauvais observants; 76% et 58,16% de ceux qui avaient respectivement trois et deux prises par jour étaient aussi de mauvais observants. Konin [4] avait trouvé 77,3% des patients ayant plus de trois comprimés par jour et 95,3% de ceux qui avaient trois prises journalières ou plus mauvais observants. L’amélioration de l’observance passerait en partie par une administration en monoprise des médicaments. Le choix doit être porté sur les associations fixes avec des molécules ayant une longue demi-vie.

## Conclusion

L’observance thérapeutique est un véritable problème auquel le couple médecin-patient est confronté. Elle a été mauvaise dans notre étude. Plusieurs facteurs peuvent l’influencer notamment l’âge, la profession et le revenu mensuel des patients, le mode de traitement et son coût.
